# 干扰素α-1b、IL-2联合沙利度胺治疗allo-HSCT后RUNX1::RUNX1T1持续阳性的伴KIT突变急性髓系白血病1例报告并文献复习

**DOI:** 10.3760/cma.j.cn121090-20250331-00160

**Published:** 2025-11

**Authors:** 瑞华 米, 琳 陈, 琳 王, 轶轩 马, 粤文 符, 旭东 魏

**Affiliations:** 郑州大学附属肿瘤医院/河南省肿瘤医院，郑州 450000 The Affiliated Cancer Hospital of Zhengzhou University/Henan Cancer Hospital, Zhengzhou 450008, China

## Abstract

回顾性分析1例伴KIT p.D816V突变的核心结合因子相关急性髓系白血病（CBF-AML）患者，阿伐替尼诱导治疗失败后桥接异基因造血干细胞移植（allo-HSCT）的治疗经过，并进行文献复习。患者，男，64岁，DA（柔红霉素+阿糖胞苷）方案诱导治疗未缓解，阿伐替尼联合DCHG（地西他滨+高三尖杉酯碱+阿糖胞苷+G-CSF）方案再次诱导治疗达完全缓解（CR）及流式细胞术微小残留病（MRD）阴性，但患者RUNX1::RUNX1T1融合基因未转阴；在巩固治疗期间，患者流式细胞术MRD转阳及融合基因水平进行性升高，后行非血缘9/10相合allo-HSCT，移植后融合基因持续阳性，后给予干扰素α-1b、白细胞介素2（IL-2）联合沙利度胺（简称“干白沙”）方案治疗（期间先后经历干扰素、IL-2两药联合且剂量增加、泊马度胺加入及“干白沙”方案常规剂量及增加剂量等药物调整），目前MRD阴性已维持5个月余，治疗期间耐受性良好。表明“干白沙”方案可为阿伐替尼治疗失败的allo-HSCT术后RUNX1::RUNX1T1融合基因持续阳性的伴KIT p.D816V突变阳性的CBF-AML患者，提供了一种新的治疗手段，且耐受性良好。

## Abstract (en)

Here we report the case of a CBF-AML patient with KIT p.D816V mutation who failed avapritinib induction therapy and subsequently underwent bridging allogeneic hematopoietic stem cell transplantation（allo-HSCT）, along with a literature review. The patient was a 64-year-old male who did not achieve remission after induction therapy with the DA regimen（Daunorubicin + Cytarabine）. After reinduction with avapritinib combined with the DCHG regimen（Decitabine + Homoharringtonine + Cytarabine + Granulocyte colony-stimulating factor）, he attained complete remission（CR）and flow cytometry minimal residual disease（MRD）negativity, but the RUNX1::RUNX1T1 fusion gene remained positive. During consolidation therapy, flow MRD reappeared, and the fusion gene level progressively increased. The patient then underwent a 9/10 HLA-matched unrelated donor allo-HSCT. Post-transplant, the fusion gene persisted, prompting treatment with the “ITI” regimen（with dose adjustments, including sequential addition of interferon and interleukin-2, pomalidomide incorporation, and standard vs. escalated dosing of “ITI” regimen）. Currently, MRD negativity has been maintained for over 5 months, with good treatment tolerance. This finding suggest that the “ITI” regimen may serve as a novel and well-tolerated therapeutic option for CBF-AML patients with persistent RUNX1::RUNX1T1 fusion gene positivity after allo-HSCT and KIT p.D816V mutation, particularly in cases of avapritinib treatment failure.

KIT突变存在于约40％的t（8;21）异常急性髓系白血病（AML）和33％的inv（16）异常的AML患者中[1]，影响核心结合因子相关急性髓系白血病（CBF-AML）的预后。阿伐替尼是一种强效、高选择性KIT和血小板衍生生长因子受体（PDGFRA）抑制剂，近年来在伴KIT突变的AML患者中，不管应用在移植前桥接移植[2]还是移植后清除微小残留病（MRD）降低复发率[3]–[4]，均获得了不错的疗效。在阿伐替尼靶向药物前时代甚至现在，降低以及预防移植后复发的主要措施包括预防性供者淋巴细胞输注（DLI）、靶向药物维持治疗及去甲基化药物等。在对于阿伐替尼治疗失败的伴KIT突变AML患者治疗的探索中，我中心应用干扰素α-1b（IFNα-1b）、白细胞介素-2（IL-2）联合沙利度胺（Thal）（简称“干白沙”）方案成功治疗1例异基因造血干细胞移植（allo-HSCT）后RUNX1::RUNX1T1融合基因持续不转阴患者，现将病例资料汇报如下。

## 病例资料

患者，男，64岁。2023年2月8日因无明显诱因下出现齿龈出血就诊于当地医院，入院后血常规示：WBC 20.26×10^9^/L，HGB 91 g/L，PLT 6×10^9^/L，骨髓象：增生极度活跃，原始细胞95％，外周血涂片示：原始细胞占84％。流式细胞术免疫分型示：异常细胞约占82.98％，主要表达CD38、CD56、CD13、HLA-DR、CD33、CD34、CD117、CD71、cMPO。融合基因检测示RUNX1::RUNX1T1阳性（未行定量检测）。染色体核型图谱分析示：45，X，−Y，der（3）t（3;14）（p12;q23），add（4）（p15），der（8）t（8;21）（q22;q22.1）t（8;17）（q11;q23），der（14）t（4;14）（p15;q23），der（17）t（8;17）（q11;q23），der（21）t（8;21）（q22;q22.1）[20]，二代基因测序（NGS）检测结果显示KIT p.D816V。诊断为“AML伴t（8;21）（q22;q22.1）/RUNX1::RUNX1T1中危组”。入院后合并消化道出血，给予止血等对症处理后于2023年2月20日予DA（具体剂量不详）方案化疗。2023年3月27日骨髓象：增生明显活跃，原始血细胞占34％，于2023年3月29日再次行CAG预激方案（具体不详）治疗。

2023年4月2日患者入我院，血常规：WBC 2.90×10^9^/L，HGB 98 g/L，PLT 229×10^9^/L，骨髓象：增生活跃，原始粒细胞36.8％，白血病MRD：CD34^+^CD117^+^CD13^+^CD33^+^CD7^-^HLA-DR^+^CD38^+^CD15^-^CD64^-^CD19^+^CD56^+^ CD45^dim+^，异常髓系原始细胞占16.03％，RUNX1::RUNX1T1融合基因/ABL（qPCR检测，灵敏度阈值为10^−5^）：264.48％。NGS结果示：KIT p.D816V（VAF:4.73％）。染色体核型图谱分析显示：46,XY，t（3;4）（p22;p15）,−8,add（17）（q24）,der（21）t（8;21）（q22;q22）[3]/46,XY[7]。明确诊断为“AML伴t（8;21）（q22;q22.1）/RUNX1::RUNX1T1中危组未缓解”，于2023年4月5日给予D-CHG联合阿伐替尼化疗，具体：地西他滨（DAC）25 mg第1～5天，静脉滴注；阿糖胞苷（Ara-C）25 mg每12 h 1次，第6～19天，皮下注射；高三尖杉酯碱（HHT）2 mg，第6～13天，静脉滴注；G-CSF 150 µg，每日2次，皮下注射；阿伐替尼100 mg，每日1次，口服。4月17日骨髓象：增生减低，原始粒细胞7.5％，RUNX1::RUNX1T1融合基因/ABL：22.02％，流式细胞术MRD：阴性。

2023年5月4日及2023年6月6日返院后复查：骨髓象CR，流式细胞术MRD均为阴性，RUNX1::RUNX1T1融合基因/ABL分别为16.94％和6.71％，分别于5月6日及6月8日予D-CHG联合阿伐替尼方案化疗（具体同我院第1周期）。

2023年7月10日返院，骨髓象示CR，RUNX1::RUNX1T1融合基因/ABL：17.46％，MRD示异常髓系原始细胞占1.31％，患者自开始应用阿伐替尼后，一直持续服用，目前MRD较前升高，考虑阿伐替尼耐药，接下来拟行allo-HSCT，移植前暂给予“干白沙”方案治疗。

2023年8月4日为行allo-HSCT返院，完善移植前相关检查，骨髓象示CR，RUNX1::RUNX1T1融合基因/ABL：13.19％，MRD检测示异常髓系原始细胞占0.53％。移植前患者体能指数评分：KPS评分：100分；ECOG评分：0分；HCT-CI评分：0分，遂于8月4日给予DAC 10 mg/d移植前桥接治疗，8月7日始给予MeCBA方案预处理，具体：司莫司汀（Me-CCNU）250 mg/m^2^，−7 d；阿糖胞苷（Ara-C）2.0 g·m^−2^·d^−1^，−6～−2 d；克拉屈滨（Cla）5 mg·m^−2^·d^−1^，−6～−2 d；白消安（Bu）3.2 mg·kg^−1^·d^−1^，−6～−3 d，兔抗人胸腺细胞免疫球蛋白（r-ATG）2 mg·kg^−1^·d^−1^，−4～−1 d。2023年8月14日回输非血缘HLA 9/10相合外周血干细胞，受者血型O型Rh（D）阳性，供者血型B型Rh（D）阳性。回输有核细胞7.49×10^8^/kg，CD34^+^细胞6.22×10^6^/kg，+12 d粒细胞植入。2023年9月11日骨髓象：增生活跃，原始粒细胞0％，MRD：阴性，RUNX1::RUNX1T1融合基因/ABL：0.18％，c-KIT突变示阴性，染色体核型图谱分析显示：46,XY[20]，供者细胞嵌合率（STR）为98.13％，患者血象恢复出院。

2023年9月17日因“尿痛、排尿困难”入院，考虑移植后合并出血性膀胱炎，给予对症处理后好转。2023年10月11日骨髓形态学：增生明显活跃，原始粒细胞0.4％，RUNX1::RUNX1T1融合基因/ABL：0.057％，流式细胞术MRD：阴性，STR为98.33％，提示原发病病情稳定。

2023年11月10日骨髓象示：增生明显活跃，未见原始粒细胞，RUNX1::RUNX1T1融合基因/ABL:0.43％，流式细胞术MRD：阴性，STR为99.56％，提示融合基因较前升高，于2023年11月15日停用抗排异治疗药物。

2023年12月8日返院，血常规：WBC 4.61×10^9^/L，HGB 107 g/L，PLT 104×10^9^/L。骨髓形态学：增生活跃，未见原始粒细胞，RUNX1::RUNX1T1融合基因/ABL：0.15％，流式细胞术MRD：阴性，STR为99.2％，全身无明显移植物抗宿主病（GVHD），遂给予IFNα-1b 60 µg隔日1次共5次、IL-2 200万IU隔日1次共5次（两药隔天交叉应用）。患者移植后不同时间节点的RUNX1::RUNX1T1融合基因和嵌合体变化情况见图1。

**图1:**
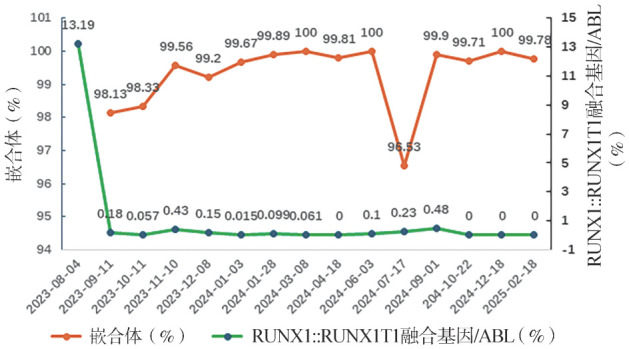
患者移植后不同时间节点的RUNX1-RUNX1T1融合基因和嵌合体变化情况 **注** 2024年4月18日融合基因示阴性，但因此次结果内参基因拷贝数较低，考虑为假阴性

患者自2023年9月11日以来，血常规示三系均正常，流式细胞术MRD均阴性。自2024年1月3日以后清除MRD的具体用药情况：2024年1月3日至2024年4月18日：IFN α-1b 60 µg隔日1次、IL-2 200万IU隔日1次（两药隔天交叉应用，其中第1个月共5次，第2个月共7次，第3个月共10次）；2024年4月18日至2024年6月3日：IFNα-1b 60 µg隔日1次、IL-2 200万IU隔日1次（两药为同一天应用）联合Thal 100 mg每晚1次；2024年6月3日至2024年9月1日：IFNα-1b 60 µg隔日1次、IL-2 200万IU隔日1次、泊马度胺4 mg每晚1次；2024年9月1日至2024年12月18日：IFNα-1b 60 µg每日1次、IL-2 200万IU每日1次、Thal 100 mg每晚1次（因经济的原因调整）；2024年12月18日至2025年2月18日：IFNα-1b 60 µg隔日1次、IL-2 200万IU隔日1次、Thal 100 mg每晚1次。

截至末次随访时，患者移植后仅出现一过性出血性膀胱炎症状，余未见发热、皮疹、肌肉酸痛、腹泻、肝功能损伤等不适，耐受性良好，目前在持续治疗中。

## 讨论及文献复习

复发是影响移植后AML患者长期生存的关键问题，其中KIT突变是CBF-AML患者移植后复发的高危因素[5]。研究表明，监测MRD可以有效预测移植后急性白血病复发[6]。对CBF-AML患者而言，当移植后出现分子生物学阳性时，应及时采取措施，降低疾病复发风险。目前预防移植后复发的措施主要包括根据病情早期调整免疫抑制剂、免疫治疗（包括预防性DLI、干扰素、IL-2等）、靶向药物维持治疗及去甲基化药物等。

对allo-HSCT后MRD阳性的t（8;21）AML患者，抢先给予干扰素α和DLI，2年累积复发率仍分别为55.6％和31.4％[7]。对于无法实施DLI的患者，符粤文团队于2019年将干扰素联合IL-2应用于allo-HSCT后高危及MRD升高的患者，总体有效率为73％，GVHD总发生率为63％，其中急性GVHD发生率为10％，慢性GVHD发生率为52％，均为局限型慢性GVHD，不仅未影响患者的正常生活，而且长期维持移植物抗白血病效应，降低患者的复发率，维持长期生存[8]。本例患者移植后分子生物学持续阳性，起始给予干扰素联合IL-2分别隔日应用，每月依据耐受情况，将两种药物逐渐加量，治疗3个月，患者融合基因仍未转阴。

2019年我团队应用“干白沙”方案干预治疗MRD阳性AML，总有效率为72.2％[9]。2021年我团队将“干白沙”方案应用于RUNX1::RUNX1T阳性的AML患者，总有效率为90％[10]。2021年我团队观察其三药联合的协同机制，发现“干白沙”方案可提高外周血CD4^+^/CD8^+^比例及NK细胞比例，增加NK细胞Granzyme-B及Perforin的表达，促进IFN-γ的产生并减少血管内皮生长因子（VEGF）的分泌，这可能增强AML患者机体抗肿瘤能力[11]。

目前尚未见“干白沙”方案应用于接受allo-HSCT的患者。我们将该方案应用于本例患者，但“干白沙”方案应用1个周期后，患者融合基因持续阳性。

泊马度胺为第3代免疫调节药物（IMiD），其在多发性骨髓瘤中的应用较为普遍，且疗效和安全性均优于沙利度胺。但其在AML中的应用相对较少，2020年国外学者[12]将泊马度胺应用于AML患者，亚组分析显示：使用泊马度胺治疗的高危核型AML患者CR/CRi达86％，CR的实现与CD8^+^终末分化效应记忆细胞（TEMRA）的显著降低有关，而诱导化疗无反应者这群细胞保持不变或增加。故我们将干扰素α-1b、IL-2联合泊马度胺组成“干白泊”方案应用于本例患者，但“干白泊”方案应用2个周期后，患者融合基因不但没下降，反而进行性升高，后因经济原因暂停泊马度胺治疗。

关于度胺类药物在AML中的疗效分析及机制探讨数据较少，2024年崔玉等[13]研究了三种IMiD抗AML的作用机制，发现三种IMiD药物均可抑制AML细胞的增殖，促进AML细胞凋亡，且来那度胺、泊马度胺的作用明显强于沙利度胺，这种作用可能与上调AML细胞中CRBN的表达，下调CK1a、IKZF1、VEGFA的表达有关；三种IMiD药物均能够显著增强T、NK和DC细胞的免疫功能，其中泊马度胺和来那度胺的作用明显强于沙利度胺。目前更缺乏三种免疫调节药物在临床中的直接对比研究。

在2019年我团队[9]在“干白沙”清除AML MRD研究中发现，常规剂量“干白沙”治疗无效的3例患者中，再次接受加量的“干白沙”方案，其中2例MRD转阴，1例MRD水平下降。故本例患者接下来给予加量的“干白沙”方案，1个月后融合基因转阴，目前已维持阴性5个月余，“干白沙”方案在逐渐减量中，整个清除MRD治疗期间，患者耐受性良好，未见发热、皮疹、肌肉酸痛、腹泻、肝功能损伤等不适。本例患者融合基因的转阴，可能与“干白沙”方案加量后患者免疫功能进一步增强有关，很遗憾的是未对患者用药前后的免疫功能进行检测。该患者的持续疗效和安全性尚有待进一步观察。

另外，从治疗的方案选择来看，该患者仅是MRD的持续阳性，目前尚无适合的临床试验供选择，包括新药临床试验甚至CAR-T治疗（后者目前尚无针对髓系肿瘤的商业化产品），并且家庭已无足够的经济能力承受二次移植；从治疗的经济学考虑，这三个药物均为集采产品，每个月的治疗费用相对较低。

总之，本研究显示“干白沙”方案干预治疗allo-HSCT后RUNX1::RUNX1T1融合基因持续阳性的患者初步有效，但治疗期间在监测疗效的同时应严密观察患者GVHD的发生情况。本研究对伴KIT突变CBF-AML患者，阿伐替尼治疗失败后提供了可能再次逆转MRD的方法，但因是个例报告，随访时间相对较短，还需要进一步的大规模和前瞻性研究，并进行长期随访。
